# Role of Coffee Caffeine and Chlorogenic Acids Adsorption to Polysaccharides with Impact on Brew Immunomodulation Effects

**DOI:** 10.3390/foods10020378

**Published:** 2021-02-09

**Authors:** Cláudia P. Passos, Rita M. Costa, Sónia S. Ferreira, Guido R. Lopes, Maria T. Cruz, Manuel A. Coimbra

**Affiliations:** 1 LAQV-REQUIMTE, Department of Chemistry, University of Aveiro, 3810-193 Aveiro, Portugal; rita.mendes.costa.14@gmail.com (R.M.C.); soniasferreira@live.ua.pt (S.S.F.); guido@ua.pt (G.R.L.); mac@ua.pt (M.A.C.); 2 CICECO—Aveiro Institute of Materials, Department of Chemistry, University of Aveiro, 3810-193 Aveiro, Portugal; 3 Center for Neuroscience and Cell Biology, University of Coimbra, Azinhaga de Santa Comba, 3004-517 Coimbra, Portugal; trosete@ff.uc.pt; 4 Faculty of Pharmacy, University of Coimbra, Pólo das Ciências da Saúde, Azinhaga de Santa Comba, 3000-548 Coimbra, Portugal

**Keywords:** espresso coffee, soluble coffee, melanoidins, caffeine, chlorogenic acids, polysaccharides, macrophages, inflammation

## Abstract

Coffee brews have High Molecular Weight (HMW) compounds with described immunostimulatory activity, namely polysaccharides and melanoidins. Melanoidins are formed during roasting and are modified during brews technological processing. In addition, brews have Low Molecular Weight (LMW) compounds, namely free chlorogenic acids and caffeine, with well-known anti-inflammatory properties. However, this study shows that both espresso and instant coffee brews did not present immunostimulatory neither anti-inflammatory in vitro activities. It is possible that the simultaneous existence of compounds with antagonistic effects can mitigate their individual effects. To test this hypothesis, an ultrafiltration separation process was applied, studying the behavior of coffee brews’ HMW on retention of LMW compounds. Several ultrafiltration sequential cycles were required to separate retentates from LMW compounds, suggesting their retention. This effect was higher in instant coffee, attributed to its initial higher carbohydrate content when compared to espresso. Separation of HMW and LMW compounds boosted their immunostimulatory (6.2–7.8 µM nitrites) and anti-inflammatory (LPS induced nitrite production decrease by 36–31%) in vitro activities, respectively. As coffee anti-inflammatory compounds are expected to be first absorbed during digestion, a potential in vivo fractionation of LMW and HMW compounds can promote health relevant effects after coffee intake.

## 1. Introduction

As one of the most popular and consumed beverage in the world, coffee is constantly a subject of scientific study, namely for the identification of the compounds responsible for coffee consumption associated health effects [1,2,3,4,5,6,7,8,9]. One theme of great importance on coffee consumption is its relation to the inflammation process as coffee contains multiple substances that individually are known to impact inflammatory markers [10]. On one hand, caffeine and chlorogenic acids (CGA, the most representative source of phenolic compounds in coffee) have reported anti-inflammatory properties [11,12,13]. On the other hand, the immunostimulatory potential of coffee has been attributed to polysaccharides, more specifically a pro-inflammatory potential associated to coffee infusion galactomannans [14] and instant coffee arabinogalactans [15,16]. Nevertheless, arabinogalactan-proteins have been described to have the ability to induce the production of both immunostimulatory and anti-inflammatory cytokines [15].

Polysaccharides are the compounds in higher abundance in coffee beverages, contributing to viscosity and thickness of coffee brews [17,18]. While galactomannans are associated to the viscosity of the liquid phase, the arabinogalactans, despite their high molecular weight, show low viscosity properties. Therefore, when carbohydrates are address as important to the foam stability in espresso coffee, this effect is mainly associated to the presence of galactomannans [17]. In commercial instant coffee, to facilitate the industrial processing and to allow a higher soluble solids content before drying, viscosity is usually decreased by the use of mannanase treatments, converting galactomannans into galactomannan-oligosaccharides [19]. As the pre-processing steps can contribute to modify carbohydrates structure, these steps can also affect structure-function related properties.

Caffeine and CGA are coffee low molecular weight compounds included in coffee, which can be recovered in the low molecular (LMW) material fractions. Coffee brews also contain CGA associated with polysaccharides and proteins, chemically bounded in complex structures recovered in the high molecular weight (HMW) material. These phenolic compounds (caffeic, ferulic, and p-coumaric acids esterified with quinic acid) are present in the HMW mainly in condensed form [20], with a small portion of phenolic compounds in ester linked-form, eventually forming melanoidins [21]. Generically, melanoidins are described as complex high molecular weight nitrogenous brown colour compounds formed during roasting and defined as end-products of Maillard reaction [2,8,22].

Compiling the individual activities of coffee components may be the simplest way to evaluate the effect of a complex matrix such as coffee. However, considering the existence of antagonistic/synergistic effects, these may at the same time attenuate or boost the individual activities determined. For example, the presence of flavonoids potentiate the anti-inflammatory activity of caffeoylquinic acid [23]. Likewise, taking into account the anti-inflammatory properties of the compounds present in coffee LMW and the immunostimulatory activity of coffee polysaccharides in HMW material, the impact of their coexistence in the coffee brew needs consideration. For this, well-purified and characterized fractions should be obtained for in vitro evaluation of the individual activities of coffee components for comparison with the activities of the brews. Nevertheless, due to the existence of adsorption phenomena between LMW and HMW compounds, purification methods that assure this purpose need to be applied. 

As different types of brew preparations can have a distinct composition, in this study, espresso coffee and instant coffee were used to obtain LMW and HMW purified fractions with known composition. To disclose the impact of the coexistence in coffee brews of compounds with immunostimulatory and anti-inflammatory activities, the purified fractions as well as the brews were evaluated on an in vitro model to access these activities.

## 2. Materials and Methods

### 2.1. Samples Preparation

Three espresso coffee samples were obtained in a local supermarket from commercial batch of single-dose coffee capsules commercialized in Portugal, which uses to be a mixture of Arabica and Robusta varieties. These powders were respectively used to prepare espresso coffee 1, espresso coffee 2, and espresso coffee 3 brews. A total of 10 individual espresso coffee samples (40 ± 2 mL) were prepared on a Delta Q^®^ (QOSMO Nacré model, Portugal) machine by extraction with tap water, then, the 400 mL were mixed. This sample was named espresso coffee 1. For characterization, an aliquot of 4 mL was collected, frozen, and freed-dried. The remaining solution was further filtered, removing any suspended particle that could interfere with the ultrafiltration process, and dilute to a total volume of 500 mL. The second sample, espresso coffee 2, was chosen to show the reproducibility of the process repeating the brewing process from 10 commercial single-dose coffee samples (40 ± 2 mL). A third espresso coffee sample was also prepared with 25 single-dose coffee capsules, and named as espresso coffee 3.

Three instant coffee powder samples were obtained in a local supermarket from commercial batches. These powders were respectively used to prepare instant coffee 1, instant coffee 2, and instant coffee 3 brews. Instant coffee 1 was prepared from 15 g of instant coffee powder dissolved in 500 mL of distilled water at 80 °C and stirring during 10 min at 80 °C. The solution was then cooled down and allowed to settle down at 4 °C during 48 h, removing the insoluble solids by decantation and filtration. For characterization, an aliquot of 4 mL of the filtrate was collected, frozen, and freed-dried. A second instant coffee sample (instant coffee 2) was prepared using a higher amount of coffee powder (31.5 g), from a different brand, dissolved in the same volume of 500 mL of distilled water at 80 °C [16]. The preparation of the brew was the same as described for instant coffee 1. A third sample, from a different coffee sample brand (instant coffee 3), was prepared with 10 g of instant coffee powder dissolved in 500 mL of distilled water at 80 °C. The preparation of the brew was the same as described for instant coffee 1.

### 2.2. Coffee Samples Ultrafiltration

**Figure 1:**
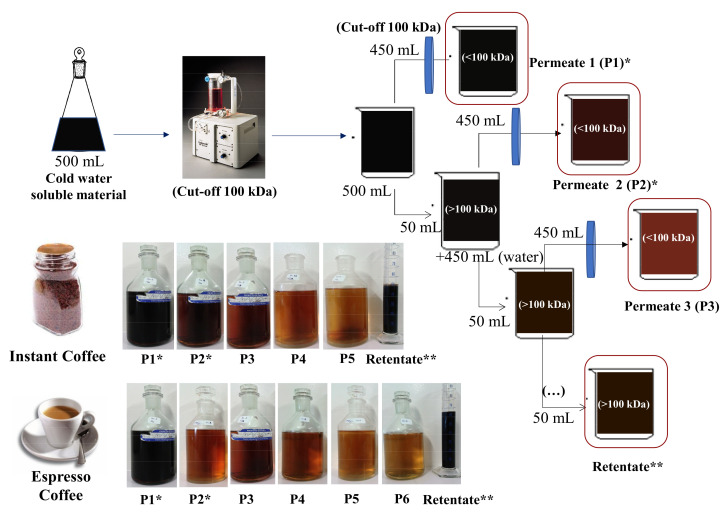
Schematic representation of the ultrafiltration process with photos of the successive Permeates and Retentate for espresso coffee and instant coffee brews. * Permeates 1 and 2 represent the most abundant fractions of Low Molecular Weight (LMW) compounds. ** Retentate represents the fraction of High Molecular Weight (HMW) compounds.

To separate the high molecular weight (HMW) material from the low molecular weight (LMW) material, all samples were individually submitted to a fractionation procedure by ultrafiltration (Figure 1). The ultrafiltration occurred on an ultrafiltration module—Labscale TFF System (Millipore), operating at room temperature.

Using a pellicon XL ultrafiltration module ultracel membrane with cut-off 100 kDa, and working among 10 and 20 psi transmembrane pressures, the initial 500 mL of solution in the reservoir was concentrated to 10% of the initial volume (50 mL, Retentate 1). The permeated solution was recovered, concentrated in a rotary evaporator at 40 °C, frozen and freeze-dried in a freeze drier (−50 °C, 0.1 mbar) (SCANVAC CoolSafe 9 L, Labogene, Allerød, Denmark) (Permeate 1). The ultrafiltration process was repeated by adding distilled water to Retentate 1 reaching a volume of 500 mL in the reservoir. The ultrafiltration process re-started until the solution in the reservoir was down to 50 mL (Retentate 2). The permeated solution (Permeate 2) was recovered as described for Permeate 1. The ultrafiltration process was repeated for several cycles until the conductivity of the last permeate was below 75 µS·cm^−1^. A total of 6 and 5 permeate fractions were recovered, respectively, from expresso coffee 1 and instant coffee 1 samples. For analytical purposes, an aliquot of 4 mL was collected from each Retentate, representing each cycle of the ultrafiltration sequential process. All fractions obtained were freeze-dried (−50 °C, 0.1 mbar) (SCANVAC CoolSafe 9 L, Labogene, Allerød, Denmark) and stored under an anhydrous atmosphere.

To disclose possible adsorption phenomena during the ultrafiltration process, an experiment was performed under diluted conditions, dissolving 0.50 g of caffeine and 1.0 g of the final Retentate in 500 mL of water.

### 2.3. Characterization of Brews and Ultrafiltration Permeates and Retentates

#### 2.3.1. Sugar Analyses

The individual neutral sugars were determined after acid hydrolysis, derivatization to alditol acetates, and analysis by GC-FID [24]. The polysaccharides were hydrolysed using 2 M sulfuric acid at 120 °C during 1 h. 2-deoxyglucose was added as internal standard and the monosaccharides were reduced with NaBH_4_ and acetylated with acetic anhydride in the presence of 1-methylimidazole. Alditol acetate derivatives were separated with dichloromethane and analysed by GC (Clarus 400 chromatograph, PerkinElmer, Waltham, MA, USA) equipped with a FID detector and a DB-225 column (30 m × 0.25 mm and 0.15 µm of film thickness). Sugars were determined in duplicate.

#### 2.3.2. Caffeine and Free Chlorogenic Acids (CGA) Quantification

Aliquots of 10 g/L of each fraction were prepared and characterized for their caffeine and CGA content [25]. The aliquots were filtered using 0.45 µm filters prior HPLC-DAD injection. A HPLC-DAD (PerkinElmer, Waltham, MA, USA) apparatus was equipped with a C18 column (Sherisorb S10 ODS2 (Waters, Milford, MA, USA), 4.6 mm × 250 mm, 10 µm) and a UV–Vis-Dionex Ultimate 3000 (PerkinElmer, Waltham, MA, USA) detector. The runs were equilibrated with formic acid 5% (eluent A) and eluted with a flow rate of 0.8 mL/min using methanol as eluent B and a gradient program. Caffeine was quantified by using a calibration curve made with pure caffeine (0.02–0.92 g/L). Quantification of total CGA was performed using a standard curve made with 5-cafeoylquinic acid (Sigma-Aldrich, St. Louis, MO, USA) (0.10–0.58 g/L) and expressed as 5-cafeoylquinic acid equivalents.

#### 2.3.3. Protein Estimation

The nitrogen content was determined by elemental analysis in the initial and retentate fractions in a CHNS-932 (LECO, Geleen, The Netherlands) elemental analyser with a TDC detector (2 replicates per blend). Nitrogen content was converted in protein content using a factor of 5.5, in accordance with Dumas method [26]. This procedure was only used in the samples where all alkaloids have been removed.

#### 2.3.4. Specific Extinction Coefficients Determination

To determine the specific extinction coefficients at 280 nm, 325 nm and 405 nm, (respectively *K*_mix,280 nm_, *K*_mix,325 nm_ and *K*_mix,405 nm_) about 1.0 g/L of each lyophilised fraction was solubilized in distilled water [16,26]. Several dilutions (between 1 and 500 mg/L) were then prepared from the sample. The absorptions at 280 nm, 325 nm, and 405 nm were measured in a double beam ultraviolet–visible (UV/Vis) spectrophotometer (Lambda 35, Perkin-Elmer, Waltham, MA, USA). Each curve contained at least 5 measurements with two replicate curves prepared for each sample.

#### 2.3.5. Melanoidin Browning Index (MBI)

The melanoidin browning index (MBI) was estimated by the ratio of the brown color measurement (*K*_mix,405 nm_ quantification) and the unknown material (estimated by difference to the identified polymeric material) [16,27].

### 2.4. Immunostimulatory and Anti-Inflammatory Properties in Macrophages Cell Lines

The different samples and fractions were solubilized in sterile phosphate-buffered saline (PBS) with 2% (*v/v*) dimethyl sulfoxide (DMSO) and filtered through cellulose acetate 0.22 µm sterile syringe filter (Frilabo, Maia, Portugal), under sterile conditions. The solutions were then diluted to achieve 228–330 µg/mL of sample/fraction in the culture medium, with a final concentration of DMSO lower than 0.1% (*v/v*).

Raw 264.7 cells, a mouse leukemic monocyte macrophage cell line from American Type Culture Collection (ATCC TIB-71), were cultured in Dulbecco’s Modified Eagle Medium (DMEM, A13169050, Applichem, Darmstadt, Germany) supplemented with 10% non-inactivated fetal bovine serum (Alfagene, Carcavelos, Portugal), 100 U/mL penicillin, 100 μg/mL streptomycin, and 17.95 mM sodium bicarbonate suitable for cell culture (Sigma, St. Louis, MO, USA), at 37 °C in a humidified atmosphere of 95% air and 5% CO_2_. Along the assays, morphological changes of cells were monitored by microscopic observation.

Raw 264.7 cells (0.3 × 10^6^ cells/well) were plated in a microplate of 48 wells and allowed to stabilize for 12 h. After this time, cells were either maintained in culture medium (control +) or pre-incubated with different concentrations of sample for 24 h. In the case of anti-inflammatory activity, after 1 h of incubation in culture medium (control −) or pre-incubated with different concentrations of sample for 1 h, the cells were later activated with 1 μg/mL lipopolysaccharides (LPS from *E. coli*, serotype 026:B6, Sigma Chemical Co., St. Louis, MO, USA) for a total of 24 h. Cell viability was assessed using 3-(4,5-Dimethylthiazol-2-yl)-2,5-diphenyl tetrazolium bromide (MTT, Acros Organics, Geel, Belgium) reduction colorimetric assay [28], as previously reported [29]. The production of nitric oxide (NO) was measured by nitrite accumulation in the culture supernatants, using a colorimetric reaction with the Griess reagent and a standard curve made with 0.25–50 μM NaNO_2_ [30,31].

### 2.5. Statistical Analysis

Data were analyzed with GraphPad Prism 5.01 software (OriginLab Corporation, Northampton, MA, USA, trial version). The significance of the difference was evaluated with one-way ANOVA followed by Tukey’s multiple comparison test at the significance level of 5% (α = 0.05).

## 3. Results and Discussion

### 3.1. Preparation of HMW and LMW Fractions

Espresso and instant coffee brews were ultrafiltered allowing to obtain 10% of the initial volume as high molecular weight (HMW) compounds in the Retentate solution. The Retentate of each coffee sample (Table 1) represented 9.7% of total solids for espresso coffee (espresso coffee 1) and 8.5% for instant coffee (instant coffee 1) samples, after 6 and 5 consecutive washing cycles of ultrafiltration. Along the ultrafiltration procedure, the Retentate solution became darker than the initial brews, whereas the successive Permeates became lighter brown (Figure 1). To estimate the colour of the Retentate, the *K*_mix_ coefficient was estimated for 405 nm, which is associated to brown coloured compounds [26]. A value of 0.5 was determined for the brew of both coffees and values of 1.1 and 1.4 were determined for the Retentate of espresso coffee 1 and instant coffee 1, respectively (Table 1). This increase was in accordance with the observed darker colour of the Retentate, which is characteristic of the increase of melanoidins concentration in the HMW material recovered in the Retentate of both brews.

The low molecular weight (LMW) compounds were recovered from the 450 mL of permeate solution (Permeate 1), as well as from all other permeated solutions obtained after the addition of washing water, 450 mL at each time, for several times. The permeates (<100 kDa) accounted for 90.3% and 91.7% of the total mass of espresso and instant coffee, respectively. Knowing that 78.4% of total mass of the same espresso coffee brand is lower than 12 kDa [32], it can be estimated that only 11.9% of compounds were in the range 12–100 kDa. Comparable results were obtained from a different blend (espresso coffee 2, Table 2), where the HMW (>100 kDa) compounds accounted for 3.6% of the espresso coffee soluble solids, 14.3% were in the range of 12–100 kDa, and 78.5% were <12 kDa. For the instant coffee 1 sample, 90.3% of the material was recovered as LMW and 9.1% as HMW (Table 1). Such results are in accordance with literature data provided for a different instant coffee brand, with 48.0% recovered as LMW (<10 kDa), 24.1% in the range 10–100 kDa, and 8.0% as HMW material [16]. The use of instant coffee 2 (Table 2), resulted in a total recovery of 12.3% of HMW total solids in the Retentate, which was comparable to the other espresso and instant coffee 1 samples. However, the 50% higher amount of coffee powder used to prepare this brew when compared to the first instant coffee resulted in a higher number of purification steps, from 5 to 9.

At the end of the first ultrafiltration stage, 73% of total soluble solids were recovered in Permeate 1 for espresso coffee 1 and 71% for instant coffee 1 (Table 1). A similar trend of 69% of recovery in Permeate 1 was obtained after submitting espresso coffee 2 to the same ultrafiltration process (Table 2). The use of the higher amount of solids in instant coffee 2 reduced the recovery of total soluble solids to only 17% in Permeate 1 (Table 2). Considering that the volume of Permeate 1 was 90% of the starting volume, it would be expected a recovery higher than the 71–73% total soluble solids yield, recovered from the brew by ultrafiltration. The lowest amount recovered in Permeate 1 for instant coffee 2 showed that the higher initial solids concentration had a negative impact on the recovery of LMW compounds in Permeate 1, preventing their diffusion.

### 3.2. Coffee Brews Potential Immunomodulatory Activity

The coffee brews (espresso coffee 1 and instant coffee 1) and their purified HMW material, recovered in the Retentates, as well as the LMW material recovered in Permeates 1 and Permeates 2, were used to evaluate cells viability using the in vitro MTT assay. At least 80% cells viability was observed for coffee brews and derived samples (Figure S1), a value that allows to confirm the non-toxicity of the samples under the concentrations used [33].

**Figure 2:**
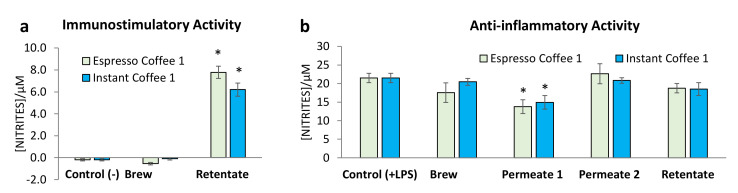
Murine macrophages nitrite production of raw 264.7 cells in culture medium: (**a**) immunostimulatory conditions and (**b**) anti-inflammatory conditions, where the samples were further treated with LPS. Each value represents the mean ± SEM from at least 3 experiments (* *p* < 0.01, compared to control).

To evaluate the potential stimulatory activities, assessed by the production of nitrites, samples from espresso coffee 1 and instant coffee 1 were incubated with macrophages Raw 264.7 cells in the absence of LPS. The immunostimulatory activity was not observed in the brew (Figure 2a), like the negative control (RPMI). However, a significant activation of macrophages was observed with the contact of the compounds present in the Final Retentates, respectively 7.8 ± 0.6 µM and 6.2 ± 0.6 µM of nitrites production for espresso and instant coffee. The immunostimulatory activity of the Retentate from both samples may be related with the presence of polysaccharides, whose immunostimulatory activity has been reported for both galactomannans [14] and arabinogalactans [16,34]. However, the fact that these compounds did not promote the immunostimulatory activity of the coffee brews suggests the possible presence of compounds with an antagonistic effect in brews, not present in the Retentate. A similar effect could have been observed when a purified instant coffee sample fraction (2E) has been assayed, showing spleen B lymphocyte, bone-marrow-derived macrophages, and dendritic cells activation [34], activities not observed on the non-purified fractions [35].

After cell activation with lipopolysaccharides (LPS), the nitrite production of the cells co-incubated with the coffee brews is statistically similar to the LPS induced nitrite production (Figure 2b). Similarly, the Retentate also showed a nitrite production that allowed to infer not to have anti-inflammatory activity. However, Permeate 1 sample, from both espresso and instant coffee, showed a significant decrease in the LPS induced nitrite production, respectively, 36 and 31% decrease in relation to the control. Nevertheless, this anti-inflammatory activity of Permeate 1 was not observed in Permeate 2, although it is expected that both Permeates contain caffeine and CGA, whose anti-inflammatory activity has been reported [11,12,13].

To explain why coffee brews, as a whole, do not present the immunostimulatory activity observed for the Retentate neither the anti-inflammatory observed in Permeate 1, the composition in caffeine, free chlorogenic acids (CGA), and carbohydrates of the brews and correspondent LMW and HMW fractions, was determined.

### 3.3. Coffee Brews and Samples Chemical Characterization

#### 3.3.1. Caffeine and Free Chlorogenic Acids (CGA)

The caffeine content in espresso coffee 1 was 68 mg per g of brew soluble solids (Table 3). This value corresponds to 1.4 g of caffeine per 100 g of espresso powder, considering that 1.2 g of brew soluble solids are obtained when using 6 g of coffee powder [32]. This value is within the range of values reported for caffeine in literature for single-dose coffee capsules (1.1 to 1.8 g/100 g of coffee powder [36,37]) but also comparable to espresso brew [9]. The 52 mg of caffeine per g of brew soluble solids obtained for the instant coffee 1 were also in the accordance with the 49–52 mg of caffeine per g of brew soluble solids described in literature for instant coffee [38,39,40]. Concerning the CGA content, it was 71 mg per g of brew soluble solids (corresponding to 1.4 g/100 g of espresso coffee powder) for espresso, and 32 mg of CGA per g of brew soluble solids for instant coffee, a value comparable to literature when considering the sum of all free CGA compounds [39]. The lower content found in instant coffee (about half the content of espresso) may result from the different origin of the commercial coffees used, balanced by CGA degradation during the roasting and instant coffee processing [41], probably integrating the melanoidins structure. This can be inferred by the reported adsorption phenomena of caffeine-CGA through hydrophobically-bound π-π molecular complexes. Although this phenomenon has been reported to occur inside plant vacuoles [42], the results here presented show that it seems also to occur in the beverage. According to Table 3, the caffeine/CGA ratio was lower than 1 for espresso coffee 1, and for instant coffee was higher than 1, a result from the impact of high temperature on degradation of CGA, whereas caffeine is thermostable. The fact that both brews can show adsorption phenomenon despite the different contents of free CGA, corroborates the existence of CGA bounded to other compounds, not detected in free form compensating the difference to caffeine. These CGA, when incorporated in melanoidins, are also able to form complexes with multivalent cations (e.g., Fe^3+^) [43], contributing to coffee brew melanoidins antioxidant activity [44].

The ultrafiltered Permeate 1 of espresso coffee 1 was composed by 5.6% of caffeine and 6.3% of CGA whereas Permeate 1 of instant coffee 1 was composed by 2.6% of caffeine and 1.7% of CGA. These values, although slightly lower than those of the brews (6.8% and 7.1% for caffeine and CGA, respectively, in espresso, and 5.2% and 3.2% in instant coffee) are much richer than those obtained in Permeate 2 (0.7% and 1.0% for caffeine and CGA in espresso and 0.5% and 0.3% in instant coffee). Considering that caffeine and CGA are compounds that have been related with anti-inflammatory activity, their higher content in Permeate 1 and lower in Permeate 2 may explain the observed Permeate 1 anti-inflammatory activity but not in Permeate 2 (Figure 2).

By the 3rd ultrafiltration cycle, less than 1 mg per g of soluble solids of caffeine or CGA in both brews were recovered (supplementary material, Table S1). However, to obtain a non-detectable value in the Retentate, 6 ultrafiltration cycles were required. Complete tables including Permeates and intermediate Retentates are provided as supplementary material (Tables S1, S4 and S5). Accordingly, no caffeine and CGA were detectable in the HMW Retentates (Table 3). However, the *K*mix coefficients estimated for 325 nm (Table 1), which are characteristic of CGA, suggesting the occurrence of covalently-linked CGA, possibly as components of the melanoidins structure [21], in accordance with the CGA degradation promoted by the roasting of the coffee powder [41]. The conductivity of the brews and permeates (Table 1) allows to infer that ionic compounds (including CGA [45]), and salts present in brews were able to diffuse through the ultrafiltration membrane.

**Figure 3:**
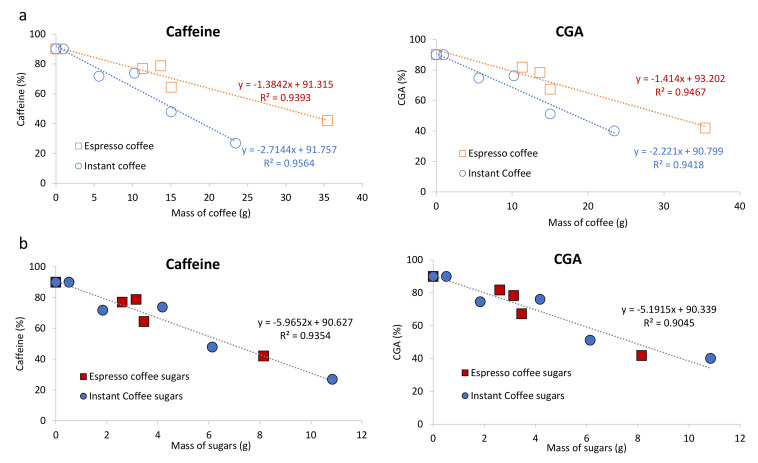
Correlations between the mass of coffee soluble solids in solution (**a**) or sugars content in the beverage (**b**) and the relative permeation by ultrafiltration from 500 mL to 50 mL of Caffeine (left) or CGA (right).

As the ultrafiltration occurs in all samples from 500 mL to 50 mL (10% of the total volume), the expected caffeine and CGA mass to be recovered should be 90% of these compounds in the initial volume. This hypothesis was proved by an experiment where caffeine and Retentate were mixed under diluted conditions, allowing the recovery of 90% of the caffeine within 90% of the initial volume. This effect was also observed for diluted coffee solutions where the amount of caffeine and CGA were monitored in both Permeate and Retentate. However, for concentrated samples, the proportion of caffeine and CGA that diffused through the ultrafiltration membrane was lower than the theoretical 90%. According to Figure 3a, the amount of caffeine (left) and CGA (right) recovered in the permeate was inversely proportional to the amount of coffee powder used. This should be due to the adsorption phenomena promoted by the HMW compounds that retain the LMW ones, as reported by Lopes et al. [32].

The adsorption effect for both caffeine and CGA was more evident when using instant coffee, about 2 times higher, when compared to espresso coffee (Figure 3a). For example, in the ultrafiltration of espresso coffee 1 (using 11.3 g of soluble solids as starting material, Table 1) allowed a recovery of 77% of the caffeine in Permeate 1 (Table 3), while in instant coffee 1 (using 15.0 g of soluble solids), the recovery of caffeine in Permeate 1 was 48% (Table 3). It is possible that the amount of carbohydrates, known to interact with coffee low molecular weight compounds may explain this behaviour [32]. In addition, this hypothesis is also reinforced by the higher concentration of carbohydrates (per g of coffee powder) known to exist in instant coffee brew when compared to espresso [46]. To test this hypothesis, the carbohydrate content in espresso coffee and instant coffee brews and derived fractions was also determined.

#### 3.3.2. Carbohydrates

The total carbohydrate content of espresso coffee 1 was 15.6% of the brew soluble solids (Table 3), a result within literature data for espresso single-dose coffee capsules [32]. A much higher content of carbohydrates was found in the instant coffee brew (40.8%), in accordance with the content found in literature for instant coffee [46]. Based on these data, it was possible to observe a correlation between carbohydrates content and the permeation of both caffeine and CGA (Figure 3b), independently of coffee brew type.

The ultrafiltration process removed all LMW compounds that could be adsorbed, allowing to obtain Retentate samples with a carbohydrate content of 43% and 52% in relation to the total soluble solids of the sample, respectively for espresso and instant coffee (Table 3). While for espresso coffee 1 this percentage was much higher than the 16% of carbohydrates content of the brew, for instant coffee 1, this value was of the same order (41% in the brew). Accordingly, Permeates 1 and 2 of espresso coffee 1 were only composed by 10 and 15% of carbohydrates, whereas Permeates 1 and 2 of instant coffee 1 were composed by 33 and 34% of carbohydrates, respectively (Table 3). These results allowed to infer the presence of more LMW carbohydrates in instant coffee 1 than in espresso coffee 1.

Mannose (Man, 45%), followed by galactose (Gal, 33%) and Arabinose (Ara, 17%), were the main sugar residues constituent of single-dose espresso coffee 1 (Table 4). On the other hand, Gal (50%), followed by Man (37%) and Ara (8%) were the main sugar residues constituent of the instant coffee brew. These results are consistent with a composition richer in galactomannans in espresso and arabinogalactans in instant coffee [16,33,46,47,48].

For espresso coffee brew and fractions, the proportion of Gal to Man ranged from 0.6–1.1. However, for instant coffee, the proportion of Gal to Man increased from 1–1.3 in the brew to 5.4 in the Retentate (Table 4). The differences in the composition of the instant coffee can be attributed to the additional processing steps required in its industrial preparation. According to literature, in a conventional instant coffee production, thermal extraction at 125 °C is followed by thermal hydrolysis at 180 °C. However, the high temperature conditions increase carbohydrates solubilization yield at expenses of carbohydrates degradation [49]. This effect does explain the higher content of carbohydrates recovered as LMW compounds in the Permeate fractions, when compared to espresso (Table 3). However, the application of thermal hydrolysis alone, would lead to the degradation of both mannose and galactose, which does not explain the high loss of mannose observed in the Retentate. Applying an endo-mannanase treatment to cleave mannans-backbone would significantly reduce coffee viscosity, an effect attributed to the presence of high molecular weight mannan-based polysaccharides, which in turn affects negatively the instant coffee technological process [50]. A direct consequence of an endo-mannanase treatment would be the degradation of mannose-polysaccharides, consistent with the low percentage of mannose recovered as HMW material during the ultrafiltration process in the instant coffee-derived retentate (Table 4).

The carbohydrate analyses showed that the amount of polysaccharides present in the brews accounted for about 4% of the total solids, whereas in Retentates this amount was 43–52% (Table 1, Table 2 and Table 3). This showed that the content of polysaccharides used in the immunostimulatory activity experiments was much lower in the coffee brews than in Retentate experiments. This fact should be considered in the interpretation of the immunostimulatory effect of these samples. Nevertheless, data reported for sample 2E by Ferreira et al. [34] showed that different concentrations of polysaccharides did not have a significant effect on the immunostimulatory activities of the fraction.

As part of the HMW compounds, melanoidins may also be contributing with their polysaccharides content towards LMW adsorption phenomena, including caffeine and CGA. Although the exact structure is still unknown [2], melanoidins presence can be estimated by melanoidins browning index (MBI). The MBI is calculated as the ratio between *K*_mix,405 nm_ and the high molecular weight compounds in the sample that are not detected as carbohydrates nor protein [16,27]. During the sequential ultrafiltration process, aliquots were recovered from the retentate reservoir and represented as intermediate retentates, allowing to determine the melanoidins content during the sequential process (Tables S4 and S5). From the intermediate retentates that contained MBI of 1.9 and 1.7, the Final Retentate achieved 2.9 and 3.5, respectively for espresso coffee 1 and instant coffee 1 samples, confirming the melanoidins enrichment as part of the HMW compounds. These melanoidins should contain modified carbohydrates [21], not accounted when carbohydrates analysis was performed.

The absence of caffeine and free CGA, as well as the enrichment in polysaccharides, independently of the composition in galactomannans or arabinogalactans, may explain the immunostimulatory activities of the Retentates observed for both brews (Figure 2a). Although the Retentates contained CGA residues linked to melanoidins, no anti-inflammatory activity was observed (Figure 2b), allowing to conclude that the bounded CGA-residues content in the melanoidins structure do not contribute to any anti-inflammatory activity, contrasting with the free CGA and caffeine present as LMW compounds in the Permeate sample 1.

## 4. Conclusions

The application of a sequential ultrafiltration scheme allowed the separation of several permeates containing low molecular weight (LMW) compounds such as caffeine and CGA. Only after several and consecutive cycles it was possible to remove the retained LMW compounds due to their adsorption to the high molecular weight (HMW) compounds. The retentate obtained was composed of polysaccharides and melanoidins. This study showed that coffee brew polysaccharides prevent caffeine and chlorogenic acids (CGA) anti-inflammatory properties in vitro, whereas caffeine and CGA present the in vitro immunostimulatory activity associated to coffee polysaccharides. Only when the LMW and HMW compounds are completely separated, the individual anti-inflammatory and immunostimulatory activities are observed. The difficulty in the separation of the two fractions is attributed to adsorption phenomena between LMW and HMW compounds, observed for both espresso and instant coffee. As phenolic compounds adsorption occurs at the level of the small intestine and coffee polysaccharides are degraded only in the colon, it can be expected that, contrarily to the in vitro assay, the individual compounds may exhibit their bioactivities in vivo.

## Figures and Tables

**Table 1:** Ultrafiltration data—mass, yield of recovery, specific extinction coefficients (*K*_mix,405 nm_, *K*_mix,280 nm_, and *K*_mix,325 nm_), and conductivity (σ) of espresso coffee 1 and instant coffee 1 brews and derived fractions.

Sample	Mass (g)	Yield (%) ^4^		*K*_mix_		σ (µS/cm)
			405	325	280	
Espresso Coffee 1						
Brew	11.3	-	0.5 ± 0.1	5.2 ± 1.1	7.1 ± 1.0	5000
Permeate 1 ^1^	7.5	72.8	-	-	-	3790
Permeate 2 ^1^	1.1	10.7	-	-	-	530
Permeate 3	0.3	2.9	-	-	-	183
Permeate 4	0.2	1.9	-	-	-	137
Permeate 5	0.1	1.0	-	-	-	93
Permeate 6	0.1	1.0	-	-	-	74
Retentate ^2^	1.0	9.7	1.1 ± 0.1	3.1 ± 0.3	4.0 ± 0.2	-
Discarded ^3^	1.0	-	-	-	-	-
Instant Coffee 1						
Brew	15.0	-	0.5 ± 0.1	3.5 ± 0.6	4.8 ± 0.8	4100
Permeate 1 ^1^	9.1	70.5	-	-	-	3240
Permeate 2 ^1^	1.8	14.0	-	-	-	432
Permeate 3	0.6	4.7	-	-	-	175
Permeate 4	0.2	1.6	-	-	-	86
Permeate 5	0.1	0.8	-	-	-	62
Retentate ^2^	1.1	8.5	1.3 ± 0.0	3.4 ± 0.3	4.1 ± 0.8	-
Discarded ^3^	2.1	-	-	-	-	-

Permeates and Retentate were recovered in accordance with Figure 1 representation. ^1^ Permeates 1 and 2 represent the most abundant fractions of Low Molecular Weight (LMW) compounds. ^2^ Retentate represents the fraction of High Molecular Weight (HMW) compounds. ^3^ Material that was discarded during filtration and ultrafiltration membrane cleaning steps. ^4^ Yield determined only considering the recovered material.

**Table 2:** Ultrafiltration data—mass, yield of recovery, specific extinction coefficients (*K*_mix,405 nm_, *K*_mix,280 nm_, and *K*_mix,325 nm_), and conductivity (σ) of espresso coffee 1 and instant coffee 1 brews and derived fractions.

Sample	Mass (g)	Yield ^4^ (%)	σ (µS/cm)
Espresso Coffee 2			
Brew	11.9	-	5150
Permeate 1 ^1^	7.7	68.8	4410
Permeate 2 ^1^	2.0	17.9	794
Permeate 3	0.5	4.5	162
Permeate 4	0.2	1.8	37
Permeate 5	0.3	2.7	26
Permeate 6	0.1	0.9	28
Retentate ^2^	0.4	3.6	-
Discarded ^3^	0.7		-
Instant Coffee 2			
Brew	31.5	-	7560
Permeate 1 ^1^	3.9	16.5	5150
Permeate 2 ^1^	6.3	26.7	2540
Permeate 3	3.4	14.4	1310
Permeate 4	2.8	11.9	820
Permeate 5	2.0	8.5	467
Permeate 6	1.4	5.9	133
Permeate 7	0.4	1.7	67
Permeate 8	0.2	0.8	50
Permeate 9	0.3	1.3	28
Retentate ^2^	2.9	12.3	-
Discarded ^3^	7.9	-	-

Permeates and Retentate were recovered in accordance with Figure 1 representation. ^1^ Permeates 1 and 2 represent the most abundant fractions of Low Molecular Weight (LMW) compounds. ^2^ Retentate represents the fraction of High Molecular Weight (HMW) compounds. ^3^ Material that was discarded during filtration and ultrafiltration membrane cleaning steps. ^4^ Yield determined only considering the recovered material.

**Table 3:** Quantification (mg/g) of caffeine, free chlorogenic acids (CGA), and carbohydrates content of espresso coffee 1 and instant coffee 1 brews, and derived ultrafiltered Permeates 1 and 2, and Retentate.

Samples ^1^	Espresso Coffee 1			Instant Coffee 1		
	Caffeine	CGA	Carbohydrates	Caffeine	CGA	Carbohydrates
Brew	67.7 ± 7.7	70.6 ± 3.4	156 ± 18	52.0 ± 8.2	32.2 ± 10.0	408 ± 0
Permeate 1	55.8 ± 1.8	62.5 ± 0.7	97 ± 21	24.9 ± 1.9	16.5 ± 1.3	328 ± 0
Permeate 2	7.0 ± 0.3	9.8 ± 0.3	145 ± 5	4.8 ± 0.6	3.3 ± 0.3	339 ± 2
Retentate	nd	nd	433 ± 4	nd	nd	517 ± 6

^1^ The remaining Permeates characterization can be found in supplementary material (Tables S1 and S2). nd—not detected.

**Table 4:** Sugars composition (mol%) for espresso coffee 1 brew, instant coffee 1 brew, ultrafiltered Permeates 1 and 2, and Retentates.

Espresso Coffee 1	Rha	Ara	Man	Gal	Glc
Brew	4.8 ± 0.2	17.9 ± 0.5	42.5 ± 2.4	31.3 ± 1.6	3.5 ± 1.0
Permeate 1	4.7 ± 0.5	20.7 ± 0.8	32.0 ± 4.2	34.6 ± 4.5	8.0 ± 0.5
Permeate 2	3.2 ± 0.2	14.2 ± 0.2	50.2 ± 3.6	27.6 ± 2.7	4.8 ± 0.4
Retentate	5.6 ± 0.4	14.0 ± 2.6	38.7 ± 0.9	40.6 ± 4.6	1.1 ± 0.1
**Instant Coffee 1**					
Brew	0.9 ± 0.2	8.4 ± 0.9	37.6 ± 2.2	50.0 ± 2.4	3.1 ± 1.2
Permeate 1	1.0 ± 0.3	9.2 ± 0.4	42.0 ± 0.9	45.3 ± 0.7	2.5 ± 0.2
Permeate 2	1.0 ± 0.0	9.0 ± 0.8	40.7 ± 2.7	47.6 ± 3.5	1.8 ± 0.0
Retentate	2.4 ± 0.1	8.5 ± 0.4	13.9 ± 0.3	74.3 ± 0.3	0.8 ± 0.3

## Supplementary Materials

The following are available online at https://www.mdpi.com/2304-8158/10/2/378/s1, Figure S1: Evaluation of macrophages viability treated with coffee samples for 24h (a) and macrophages pre-incubated with coffee samples during 1h and, further stimulation with 1µg/mL LPS for a total of 24 h (b), Table S1: Quantification (mg/g) of caffeine and free CGA during sequential fractionation of espresso coffee 1 and 2 and instant coffee 1 and 2, Table S2: Carbohydrates content and composition for single-dose capsules espresso coffee 2 initial and ultrafiltration derived Permeate fractions and Retentate, Table S3: Carbohydrates content and composition for instant coffee 3 initial and ultrafiltration derived Permeate fractions and Retentate, Table S4: Chemical characterization of Retentate fractions recovered during the sequential ultrafiltration fractionation process for espresso coffee 1, Table S5: Chemical characterization of Retentate fractions recovered during the sequential ultrafiltration fractionation process for instant coffee 1.
