# Supplementary material for: Role of Coffee Caffeine and Chlorogenic Acids Adsorption to Polysaccharides with Impact on Brew Immunomodulation Effects
Source: Foods. 2021 Feb 9;10(2):378. doi: 10.3390/foods10020378 (PMC7916192; doi:10.3390/foods10020378)
Supplement: Supplementary file 1 [file foods-10-00378-s001.pdf]

Supplementary data

Role of coffee caffeine and chlorogenic acids adsorption to polysaccharides with impact on brew immunomodulation effects

Cláudia P. Passos[1,*], Rita M. Costa[1], Sónia S. Ferreira[1], Guido R. Lopes[1,2], Maria T. Cruz[3,4], Manuel A. Coimbra[1]

[1] LAQV-REQUIMTE, Department of Chemistry, University of Aveiro, 3810-193 Aveiro, Portugal; cpassos@ua.pt, soniasferreira@live.ua.pt, guido@ua.pt, mac@ua.pt
[2] CICECO - Aveiro Institute of Materials, Department of Chemistry, University of Aveiro, 3810-193 Aveiro, Portugal
[3] Center for Neuroscience and Cell Biolgy, University of Coimbra, Azinhaga de Santa Comba, 3004-517 Coimbra, Portugal; trosete@ff.uc.pt
[4] Faculty of Pharmacy from the University of Coimbra, University of Coimbra, Pólo das Ciências da Saúde, Azinhaga de Santa Comba, 3000-548 Coimbra, Portugal
*Correspondence: cpassos@ua.pt; Tel.: + 351 234 370706.

Table of Contents

Table S1. Quantification (mg/g) of caffeine and free CGA during sequential fractionation of espresso coffee 1 and 2 and instant coffee 1 and 2. 3

Table S2. Carbohydrates content and composition for single-dose capsules *espresso coffee 2* initial and ultrafiltration derived Permeate fractions and Retentate. 4

Table S3. Carbohydrates content and composition for instant coffee 3 initial and ultrafiltration derived Permeate fractions and Retentate. 5

Table S4. Chemical characterization of Retentate fractions recovered during the sequential ultrafiltration fractionation process for espresso coffee 1. 6

Table S5. Chemical characterization of Retentate fractions recovered during the sequential ultrafiltration fractionation process for instant coffee 1. 7

Figure S1. Evaluation of macrophages viability treated with coffee samples for 24h (a) and macrophages pre-incubated with coffee samples during 1h and, further stimulation with 1µg/mL LPS for a total of 24h (b). 8

Table S1. Quantification (mg/g) of caffeine and free CGA during sequential fractionation of espresso coffee 1 and 2 and instant coffee 1 and 2.

	Caffeine	CGA	Caffeine	CGA
	Espresso Coffee 1		*Instant Coffee 1*	
Brew	67.66±7.68	70.64±3.42	51.96±8.23	32.19±9.97
Permeate 1	55.84±1.78	62.54±0.71	24.85±1.85	16.46±1.33
Permeate 2	6.95±0.29	9.82±0.28	4.76±0.61	3.39±0.31
Permeate 3	0.88±0.06	1.18±0.15	0.56±0.01	0.43±0.02
Permeate 4	0.03±0.01	0.25±0.04	0.09±0.05	0.06±0.02
Permeate 5	0.02±0.00	0.05±0.02	0.01±0.01	0.01±0.00
Permeate 6	0.01±0.00	0.02±0.01	-	-
Retentate	nd	nd	nd	nd
	Espresso Coffee 2		*Instant Coffee 2*	
Brew	61.13±1.60	67.00±2.00[b]	33.07±1.22	18.53±2.07
Permeate 1	48.14±4.36	52.48±4.51	8.89±0.69	7.42±0.67
Permeate 2	10.67±0.47	12.92±0.74	11.31±0.10	10.59±0.60
Permeate 3	1.20±0.26	1.28±0.30	6.04±4.21	5.40±0.10
Permeate 4	0.19±0.00	0.18±0.00	6.75±0.30	5.55±0.10
Permeate 5	0.04±0.02	0.02±0.00	2.89±4.30	2.42±0.40
Permeate 9	-	-	0.01±0.01	0.01±0.00
Retentate	nd	nd	nd	nd

nd – not detected.

Table S2. Carbohydrates content and composition for single-dose capsules *espresso coffee 2* initial and ultrafiltration derived Permeate fractions and Retentate.

Carbohydrate	*Espresso Coffee 2*							
**Content**	**Brew**	**Permeate 1**	**Permeate 2**	**Permeate 3**	**Permeate 4**	**Permeate 5**	**Permeate 6**	**Retentate**
**(%, w/w)**	23.0±0.0	11.5±0.7	18.5±2.1	46.5±6.0	31.5±0.7	67.0±2.1	81.0±2.0	43.0±4.1
Sugar Composition								
Rha (%)	4.2±0.3	4.5±0.2	3.2±0.0	1.9±0.3	1.9±0.1	1.4±0.2	3.0.±1.1	5.9±0.6
Ara (%)	17.2±0.1	26.0±1.4	17.4±0.3	10.2±0.9	11.5±0.8	6.6±0.5	11.7±2.4	16.9±0.3
Man (%)	44.8±0.5	31.4±2.1	47.42±0.7	65.4±3.5	62.3±5.8	68.7±1.4	50.3±9.7	39.0±1.2
Gal (%)	32.9±0.3	35.5±1.3	30.72±0.1	21.7±5.1	23.6±5.0	23.1±0.6	34.6±6.7	36.3±2.6
Glc (%)	2.2±0.2	2.9±0.5	2.6±0.7	2.7±0.8	2.2±0.2	1.8±0.1	3.2±1.5	1.9±0.6

Table S3. Carbohydrates content and composition for *instant coffee 3* initial and ultrafiltration derived Permeate fractions and Retentate.

Carbohydrate	*Instant Coffee 3*								
**Content**	**Brew**	**Permeate 1**	**Permeate 2**	**Permeate 3**	**Permeate 4**	**Permeate 5**	**Permeate 6**	**Permeate 7**	**Retentate**
**(%, w/w)**	46.3±6.0	28.5±9.2	35.5±0.7	45.5±4.9	44.5±9.2	54.0±1.4	58.5±1.2	72.3±8.3	53.3±1.4
Sugar Composition									
Rha (%)	1.3±0.0	Nd	0.9±0.6	0.8±0.2	0.8±0.0	Nd	0.7±0.3	0.6±0.2	2.8±0.2
Ara (%)	9.7±1.5	9.5±0.1	8.7±0.8	8.1±0.6	7.4±0.2	7.5±0.2	6.9±0.2	7.2±0.2	9.6±0.3
Man (%)	30.3±0.0	33.7±0.1	28.6±0.1	28.1±4.2	27.7±1.0	26.9±0.5	22.5±0.1	20.6±0.2	13.7±1.2
Gal (%)	55.1±3.4	54.2±1.2	55.8±1.8	62.0±4.9	61.5±2.1	63.1±1.0	61.0±0.5	68.2±0.3	71.7±1.7
Glc (%)	3.5±5.3	2.6±1.2	6.4±2.2	1.0±0.1	2.6±1.2	2.5±0.6	7.9±0.4	2.1±0.2	1.8±0.2

Nd – not detected.

Table S4. Chemical characterization of Retentate fractions recovered during the sequential ultrafiltration fractionation process for espresso coffee 1.

Samples	*Espresso Coffee 1*									
	K_{mix}[a]			Caffeine (%, w/w)	CGA (%, w/w)	Carbohydrate Content (%, w/w)	Nitrogen[b] (%, w/w)	Protein[c] (%, w/w)	Unknown	MBI
	405 nm	325 nm	280 nm							
Brew	0.48±0.10	5.16±1.10	7.11±1.01	6.77±0.77	7.06±0.34	15.62±1.82	-	-	-	-
Retentate 1	0.80±0.13	4.14±1.07	5.15±0.68	0.81±0.00	0.86±0.10	33.68±2.23	3.37±0.05	18.5	46.1	1.926
Retentate 2	1.03±0.25	3.60±0.66	4.54±0.88	0.52±0.06	0.13±0.01	50.05±5.48	2.80±0.01	15.4	33.9	3.539
Retentate 3	1.15±0.21	3.54±0.56	4.65±0.88	0.05±0.01	0.01±0.00	46.22±6.61	2.67±0.00	14.7	39.0	3.332
Retentate 4	1.27±0.18	3.64±0.41	4.87±0.67	Nd	Nd	41.39±8.07	2.72±0.01	14.9	43.7	3.198
Retentate 5	1.16±0.19	3.27±0.49	4.29±0.70	Nd	Nd	43.97±8.23	2.66±0.02	14.6	41.4	3.113
Final Retentate	1.13±0.12	3.14±0.28	3.99±0.22	Nd	Nd	43.30±3.86	2.65±0.00	14.6	42.1	2.893

[a]The specific extinction coefficients 405, 325 and 280 nm. [b]Nitrogen content was estimated by elemental analysis. [c]Protein content according to Dumas method (% N*5.5). Nd – not detected.

Table S5. Chemical characterization of Retentate fractions recovered during the sequential ultrafiltration fractionation process for instant coffee 1.

	Instant Coffee 1									
	K_{mix}[a]			Caffeine (%, w/w)	CGA (%, w/w)	Carbohydrate Content (%, w/w)	Nitrogen[b] (%, w/w)	Protein[c] (%, w/w)	Unknown	MBI
	405 nm	325 nm	280 nm							
Brew	0.53±0.13	3.45±0.63	4.78±0.76	5.20±0.32	3.22±1.30	40.81±0.00	-	-	-	-
Retentate 1	0.74±0.05	3.54±0.26	4.18±1.19	0.37±0.03	0.20±0.00	39.46±9.21	2.76±0.02	15.2	44.8	1.732
Retentate 2	0.92±0.09	3.25±0.28	4.09±0.63	Nd	Nd	63.73±6.40	2.33±0.03	12.8	23.4	4.181
Retentate 3	1.14±0.15	3.39±0.49	4.25±0.89	Nd	Nd	56.11±0.32	2.16±0.00	11.9	32.0	3.889
Retentate 4	1.10±0.03	3.24±0.29	4.03±0.69	Nd	Nd	64.12±2.19	2.13±0.01	11.7	24.2	4.667
Final Retentate	1.27±0.00	3.36±0.26	4.07±0.78	Nd	Nd	51.74±5.65	2.15±0.00	11.8	36.4	3.483

[a]The specific extinction coefficients at 405, 325 and 280 nm. [b]Nitrogen content was estimated by elemental analysis. [c]Protein content according to Dumas method (% N*5.5). Nd – Not detected.

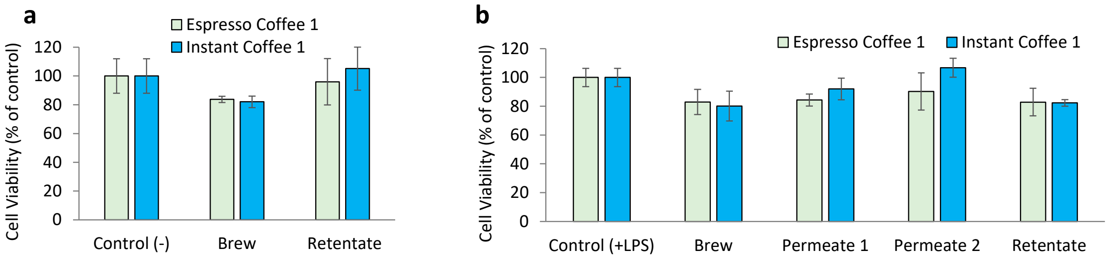

Figure S1. Evaluation of macrophages viability treated with coffee samples for 24h (a) and macrophages pre-incubated with coffee samples during 1h and, further stimulation with 1µg/mL LPS for a total of 24h (b).
